# Adult-Onset IgA Vasculitis With Concurrent Gastrointestinal and Possible Coronary Involvement

**DOI:** 10.7759/cureus.116913

**Published:** 2026-09-25

**Authors:** Maymuna Suleman, Shiva Chauhan, Antoni Chan

**Affiliations:** 1 Internal Medicine, Royal Berkshire Hospital, Reading, GBR; 2 Rheumatology, Royal Berkshire Hospital, Reading, GBR; 3 Clinical Medicine, University of Reading, Reading, GBR

**Keywords:** adult-onset iga vasculitis, cardiac involvement, coronary involvement, gastrointestinal involvement, henoch-schönlein purpura, iga nephropathy, iga vasculitis, rituximab

## Abstract

IgA vasculitis, also known as Henoch-Schönlein purpura, is a small-vessel vasculitis that can affect multiple organ systems, most commonly the skin, GI tract, and kidneys. Although it is typically a condition involving the pediatric population, adult cases may follow a more severe, rapidly progressive course with more variable presentations.

We present the case of a 50-year-old man with biopsy-proven IgA nephropathy with GI and possible coronary involvement. He initially presented with arthralgia and a purpuric rash, later developing rectal bleeding and eventually abdominal distention, nausea, and vomiting. CT imaging confirmed small bowel inflammation and ruled out mechanical obstruction. Despite treatment with oral and intravenous corticosteroids, his condition deteriorated, prompting escalation to rituximab, after which he initially improved and was discharged.

Shortly after discharge, he re-presented with chest pain and was found to have an ST-elevation myocardial infarction (STEMI) requiring critical care admission. Within days, he suffered a ventricular fibrillation arrest and passed away despite resuscitation efforts.

This case highlights the aggressive trajectory of adult-onset IgA vasculitis, the potential for severe GI involvement, the considerable diagnostic difficulty in attributing subsequent cardiac events, especially with pre-existing cardiovascular disease, and the urgent need for clearer treatment strategies in severe presentations.

## Introduction

IgA vasculitis is an autoimmune small-vessel vasculitis characterized by deposition of IgA immune complexes and activation of the downstream inflammatory cascade. It is predominantly a disease of children, but cases in adults are increasingly recognized [1]. Clinical manifestations of IgA vasculitis can include rash, fever, diarrhea, edema, and GI bleeding [1]. At present, there are no formal diagnostic criteria for adult-onset IgA vasculitis; pediatric criteria are often extrapolated to adult populations [2, 3].

IgA vasculitis is substantially less common in adults than in children, with a reported annual incidence of 0.1-1.8 cases per 100,000 adults and a median age of onset of approximately 50 years [2, 3]. Adult-onset disease is associated with a more severe clinical course and poorer renal outcomes than childhood IgA vasculitis [1, 3]. Diagnostic assessment in adults may therefore be challenging because these classification criteria were developed in pediatric populations, while adult disease may present with more variable and severe multisystem manifestations.

The European Alliance of Associations for Rheumatology/Pediatric Rheumatology International Trials Organization/Pediatric Rheumatology European Society (EULAR/PRINTO/PRES) classification criteria define IgA vasculitis by the presence of purpura or petechiae with lower limb predominance plus at least one of the following: abdominal pain, histopathological evidence of leukocytoclastic vasculitis with predominant IgA deposition, arthritis/arthralgia, or renal involvement with proteinuria/hematuria [2-5]. These criteria yield a sensitivity of 100% and a specificity of 87% [4]. Applying these criteria to our case, the presence of typical purpura (in the absence of thrombocytopenia), together with arthritis, abdominal pain, and rectal bleeding, was consistent with IgA vasculitis.

In children, IgA vasculitis is typically self-limiting with a favorable prognosis. In contrast, adult-onset IgA vasculitis tends to follow a more severe course, with higher rates of relapse, renal involvement, and long-term complications [1-3]. Management generally involves corticosteroids [1-3], with angiotensin-converting enzyme inhibitors reserved for cases of nephritis [1]. In refractory or severe cases, immunosuppressive agents such as rituximab, azathioprine, mycophenolate mofetil, or cyclophosphamide may be used, though there are no standardized treatment guidelines for adults [1].

This case highlights the diagnostic and therapeutic challenges of severe adult-onset IgA vasculitis with multisystem involvement, including life-threatening GI disease complicated by ileus and subsequent cardiac events of uncertain relationship to the underlying vasculitis. It also illustrates the limited evidence available to guide treatment escalation in severe adult IgA vasculitis, including the management of GI disease complicated by ileus and impaired oral drug absorption.

## Case presentation

A 50-year-old Caucasian man was admitted with joint pain. He had a background of biopsy-proven IgA nephropathy, seropositive rheumatoid arthritis (RA), previous gout, type II diabetes mellitus, hypertension, and heart failure with reduced ejection fraction secondary to dilated cardiomyopathy. He also had a history of splenectomy at the age of 10 for hereditary spherocytosis.

His RA had been in clinical remission, and he had discontinued his sulfasalazine for one year. A few weeks after receiving the COVID-19 vaccine, he developed arthralgia affecting multiple joints, including the shoulders and left ankle. Sulfasalazine was re-introduced, and he received a single intramuscular dose of 120 mg methylprednisolone.

Initial presentation

On Day 1, he presented to the emergency department with severe right hip pain and persistent polyarthralgia despite compliance with sulfasalazine and was unable to mobilize due to pain.Inflammatory markers were elevated, and CT imaging demonstrated fat stranding below the right hip joint without a discrete collection or destructive bony changes. Ultrasound-guided aspiration of the right hip on Day 2 was sterile and demonstrated no crystals, reducing the likelihood of septic or crystal arthritis.In the absence of other manifestations of systemic vasculitis at this stage, the presentation was initially attributed to a RA flare. Oral prednisolone 40 mg once daily was commenced on Day 3. His polyarthralgia persisted, involving the small joints of the hands, both ankles and both shoulders, and prednisolone was increased to 60 mg once daily on Day 4.

Dermatological and GI manifestations

On Day 5, one day after escalation of prednisolone to 60 mg once daily, multiple raised, reddish vasculitic papules appeared on his lower extremities, prompting consideration of systemic vasculitis.The subsequent development of GI symptoms and renal deterioration, in conjunction with the purpuric rash and polyarthralgia, strengthened the clinical diagnosis of systemic IgA vasculitis rather than an isolated RA flare. No skin biopsy was performed during this admission; therefore, the cutaneous involvement was clinically attributed to IgA vasculitis rather than histopathologically confirmed.

Despite escalating prednisolone (40 mg to 60 mg daily), inflammatory markers continued to rise. His C-reactive protein (CRP) rose from 130 mg/L to 220 mg/L. Echocardiography showed no evidence of endocarditis, and blood cultures remained negative. Vasculitis screen and viral screens, including hepatitis B virus (HBV), hepatitis C virus (HCV) and human immunodeficiency virus (HIV), were also negative. Hydroxychloroquine 200 mg once daily was commenced on Day 7 in the context of his concomitant RA and the local treatment pathway requiring prior use of two conventional disease-modifying antirheumatic drugs (DMARDs) before biologic therapy could be considered; it was not initiated as a specific evidence-based treatment for IgA vasculitis.

On Day 14, the patient developed frequent bowel motions with blood-tinged mucus and occasional dark red blood in his stools, accompanied by colicky abdominal pain and worsening renal function.Gastroenterology input was sought, and flexible sigmoidoscopy was initially planned but subsequently cancelled because of concern regarding evolving bowel obstruction. Over the following day, his GI symptoms progressed, with cessation of flatus, abdominal distension and vomiting, raising concern for ileus.

CT imaging on Day 15 demonstrated small-bowel inflammatory changes without perforation, bowel ischaemia or mechanical obstruction.GI involvement was therefore attributed clinically and radiologically to IgA vasculitis in the context of the evolving multisystem presentation; however, it was not histologically confirmed. Endoscopic assessment during the acute ileus was not pursued because of concern regarding procedural risk in the setting of suspected bowel obstruction.

Given the progression to ileus with vomiting and CT-confirmed small-bowel inflammation, oral steroids were switched to intravenous hydrocortisone (equivalent to 240 mg/day) to ensure adequate steroid absorption in the context of suspected enteric malabsorption. He was kept nil by mouth, and a Ryles tube was placed for presumed ileus secondary to IgA vasculitis. His ileus began to resolve following these measures, but he developed bloody stools once again.

On Day 16, the Renal team reviewed the patient following worsening renal function and 3+ proteinuria, with concern for active renal involvement in the context of systemic IgA vasculitis.** **This acute deterioration was interpreted clinically as possible renal involvement of the systemic IgA vasculitis episode, distinct from the patient’s pre-existing biopsy-proven IgA nephropathy; however, no repeat renal biopsy was performed during this admission.

Escalation of therapy

Despite these measures, the patient continued to deteriorate clinically and biochemically, with worsening inflammatory markers and renal function. Repeat CT excluded intussusception and mechanical obstruction. Following multidisciplinary discussion between the Rheumatology and Renal teams on Day 18, further immunosuppressive escalation was planned while pre-biologic screening was completed. Rituximab 1,000 mg intravenously was administered for a single dose on Day 21 for ongoing severe systemic IgA vasculitis despite corticosteroid therapy.

Rituximab was selected following multidisciplinary discussion on the basis of the ongoing severe multisystem disease despite corticosteroid therapy, published experience supporting its use in refractory IgA vasculitis, local clinical practice, and the patient’s background of RA. The treatment was intended to achieve systemic disease control, including the GI and renal manifestations, and was administered before the subsequent cardiac event; it was therefore not initiated for presumed cardiac involvement.

Over the week following rituximab administration, the patient demonstrated gradual clinical and biochemical improvement, including improvement in GI symptoms and renal function. Approximately one week after the first rituximab infusion, he was clinically stable with improving clinical and biochemical parameters and was discharged with a planned corticosteroid taper, close Rheumatology follow-up and a second rituximab infusion scheduled two weeks after the first dose.

Cardiac complications

**Figure 1:**
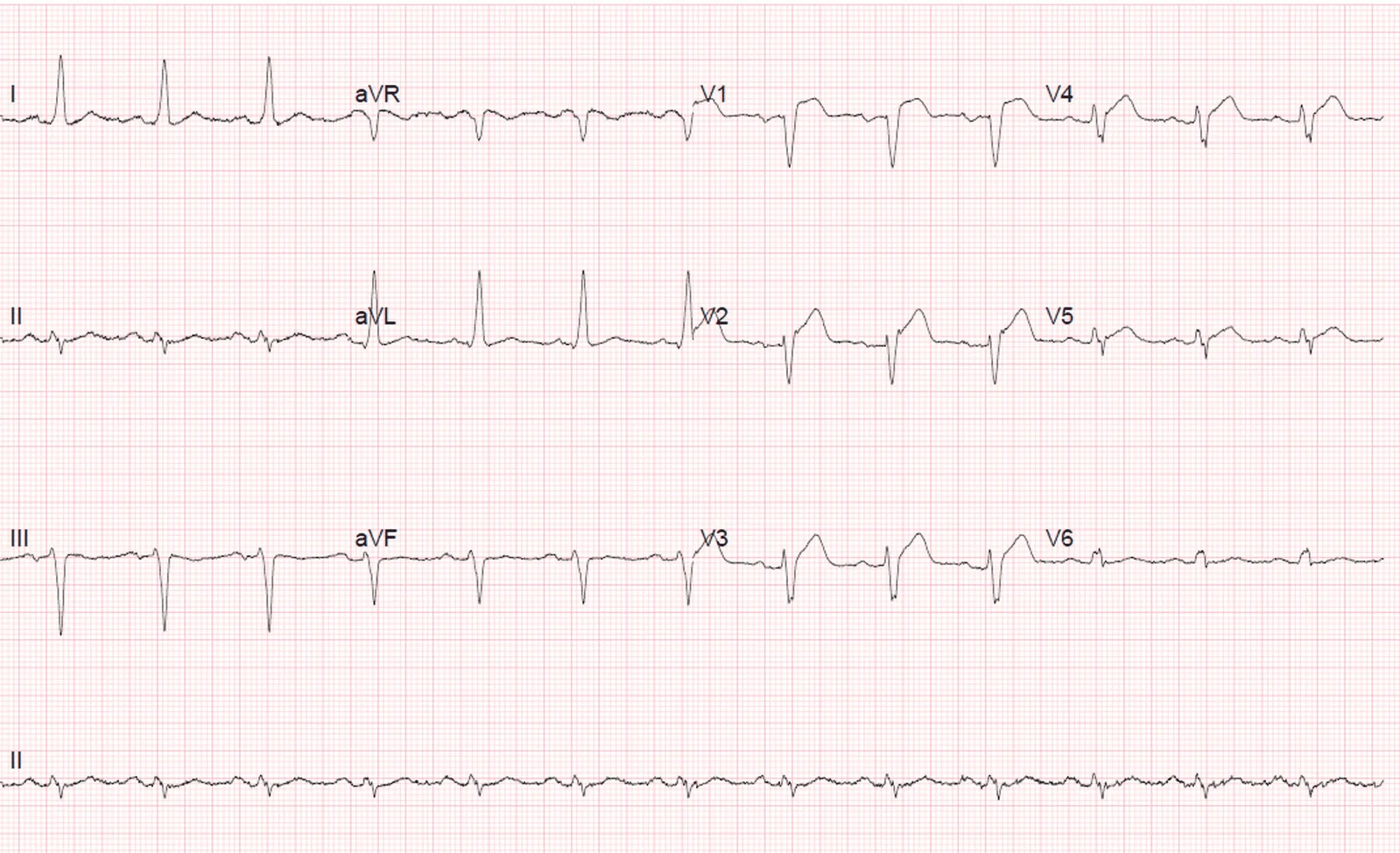
Admission ECG demonstrating anterior ST-elevation consistent with acute myocardial infarction.

**Figure 2:**
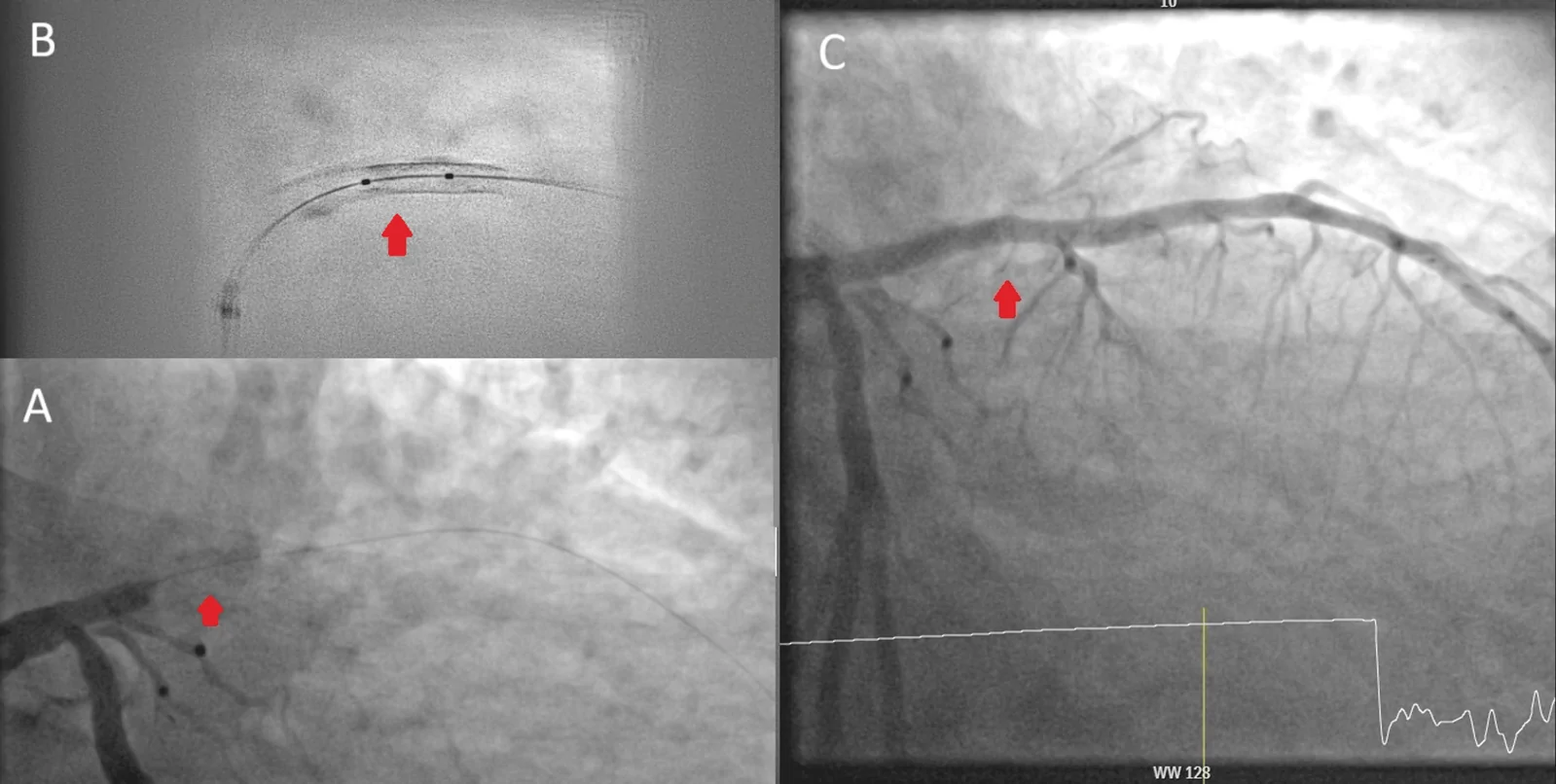
Coronary angiography findings. (A) Initial angiogram demonstrating complete occlusion of the proximal left anterior descending (LAD) artery (arrow). (B) Stent deployment across the culprit lesion (arrow). (C) Final post-deployment angiographic run confirming a patent LAD stent with restored distal flow (arrow).

One day following discharge, he re-presented with a ST-elevation myocardial infarction (STEMI). ECG had shown atypical but significant anterior ST-elevation with an elevated troponin (1705 ng/L). He was taken for percutaneous coronary intervention (PCI) with stenting of the left anterior descending (LAD) artery. Figure 1 shows his admission ECG. Figure 2 shows his initial coronary angiogram with LAD occlusion and then appearances after stenting.

**Figure 3:**
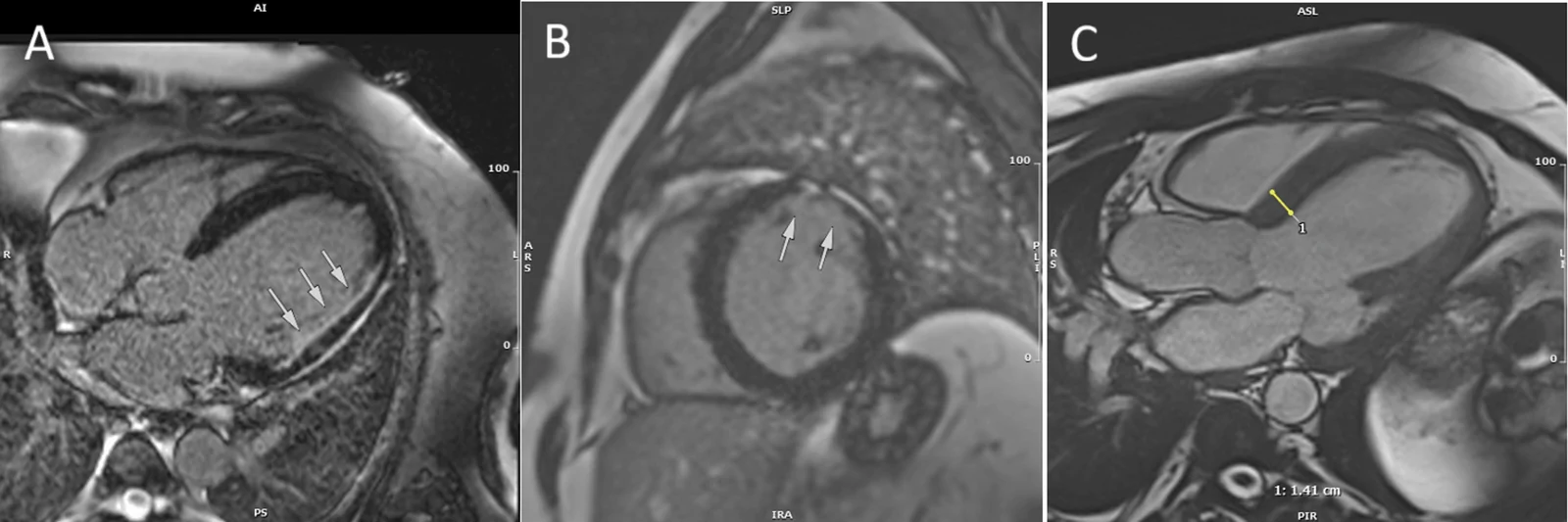
Cardiac MRI findings. (A) Post-gadolinium four-chamber view demonstrating a small area of subendocardial infarction in the anterolateral wall (arrows). (B) Post-gadolinium short-axis view confirming the same area of subendocardial ischemic injury (arrows). (C) Three-chamber view in diastole, showing mild concentric left ventricular hypertrophy (upper limit of left ventricular wall thickness is 12mm).

He had a past medical history of heart failure. Left ventricular systolic function had progressively improved with medical therapy, and recent echocardiography demonstrated an improved ejection fraction of 45%-50% from 20% a few years prior. Cardiac MRI had demonstrated a mixed pattern suggesting etiology to be predominantly hypertensive heart disease but with a focal region of subendocardial ischemic injury (Figure 3).

He was admitted to the coronary care unit, where he had suffered a ventricular fibrillation (VF) arrest. Repeat coronary angiogram demonstrated a patent LAD stent and no new abnormal angiographic findings. After one hour of resuscitation, multiple shocks, and intubation with the intensive care unit team, his rhythm and blood pressure had stabilized. He was transferred to the intensive care unit for ongoing care, where he required intubation, adrenaline and amiodarone infusions, and post-arrest critical care.

He was successfully extubated but developed fluid overload and ventilator-associated pneumonia, treated with aztreonam 2 g three times daily intravenously and linezolid 600 mg twice daily orally. He was stepped down to the coronary care unit, where he developed further melena with a hemoglobin of 56 g/L and hypotensive episodes. Esophagogastroduodenoscopy (OGD) was done, which showed only moderate duodenitis with mild diffuse oozing noted but no large ulcers or high-risk stigmata. The endoscopic findings were considered insufficient to explain the severity of the GI bleeding. The precise source and relative contribution of the underlying systemic inflammatory disease to this later bleeding episode therefore remained uncertain. High-dose intravenous omeprazole was started with a view to conducting a CT angiogram if there were further episodes of melena.

However, he suffered a further VF arrest. The recurrent ventricular fibrillation was similarly likely to have had a multifactorial substrate. Potential contributors included the recent myocardial infarction, pre-existing dilated cardiomyopathy and structural heart disease, and the associated baseline arrhythmic risk. Myocardial inflammation or microvascular involvement related to IgA vasculitis represents an additional theoretical possibility, but neither was specifically demonstrated in this patient. The recurrent ventricular arrhythmias therefore cannot be attributed to vasculitic cardiac involvement alone.

Despite advanced life support, return of spontaneous circulation was not achieved, and the patient passed away.

The chronology of the patient’s clinical progression, key investigations, treatment escalation, and subsequent deterioration is summarized in Table 1.

**Table 1:** Timeline of clinical progression, key investigations, treatment escalation, and subsequent clinical deterioration.

Date/period	Clinical progression and investigations	Management and clinical decision
Day 1	Admitted with severe right hip pain and persistent polyarthralgia, with inability to mobilise. CT demonstrated fat stranding below the right hip joint without a discrete collection or destructive bony changes. Initial differential diagnoses included septic arthritis, crystal arthropathy and rheumatoid arthritis (RA) flare.	Admitted for investigation and management of the acute inflammatory joint presentation.
Day 2	Ultrasound-guided right hip aspiration was sterile with no crystals, reducing the likelihood of septic arthritis and gout/pseudogout. In the absence of other manifestations of systemic vasculitis at this stage, symptoms were initially attributed to an RA flare.	Continued management as an inflammatory arthritis presentation.
Day 3	Persistent inflammatory polyarthralgia, subsequently involving the small joints of the hands, ankles and shoulders.	Oral prednisolone commenced.
Day 4	Persistent symptoms despite initial corticosteroid therapy.	Prednisolone increased to 60 mg once daily.
Day 5	Development of multiple raised reddish vasculitic papules over the lower limbs, shifting the clinical picture towards systemic IgA vasculitis. Inflammatory markers remained elevated, with CRP rising from 130 to 220 mg/L during this phase. Blood cultures, viral screening and vasculitis screening were negative; echocardiography showed no evidence of endocarditis.	Further investigation and management for suspected systemic vasculitis.
Day 7	Ongoing systemic inflammatory disease in the context of concomitant RA.	Hydroxychloroquine 200 mg once daily commenced as an additional immunomodulatory agent.
Day 14	Developed diarrhoea and acute kidney injury, marking progression to GI and renal involvement.	Clinical reassessment and investigation of evolving multisystem disease.
Day 15	Developed features concerning for ileus, including abdominal distension, vomiting and cessation of flatus. CT of the abdomen demonstrated small-bowel inflammatory changes without perforation, bowel ischaemia or mechanical obstruction.	Kept nil by mouth and a Ryles tube was inserted. Oral corticosteroid therapy was changed to IV hydrocortisone, equivalent to 240 mg/day, because GI involvement and ileus raised concern regarding reliable oral steroid absorption.
Day 16	Worsening renal function with 3+ proteinuria, raising concern for active renal involvement in the systemic inflammatory process.	Renal team consulted; clinical impression was an IgA vasculitis/IgA disease flare with renal involvement.
Day 18	Persistent severe multisystem disease involving the GI and renal systems despite corticosteroid treatment.	Rheumatology/Renal multidisciplinary discussion regarding further immunosuppressive escalation. Pre-biologic screening awaited.
Day 21	Continued clinical and biochemical deterioration despite preceding therapy. Repeat CT excluded intussusception and mechanical obstruction.	Rituximab 1,000 mg IV administered for ongoing severe systemic vasculitis following multidisciplinary review.
Following week	Gradual clinical and biochemical improvement was observed following rituximab, including improvement in GI symptoms and renal function.	Corticosteroid weaning planned, with a second rituximab infusion scheduled two weeks after the first dose and close Rheumatology follow-up.
Discharge	Patient was clinically stable with improving clinical and biochemical parameters at the time of discharge, approximately one week after the first rituximab infusion.	Discharged with planned corticosteroid taper, Rheumatology follow-up and second rituximab infusion.
1 day after discharge	Re-presented with acute anterior ST-elevation myocardial infarction (STEMI). Troponin was 1,705 ng/L and coronary angiography demonstrated complete proximal LAD occlusion.	Underwent percutaneous coronary intervention (PCI) and left anterior descending (LAD) stent insertion, with restoration of coronary flow.
Subsequent CCU/ICU course	Developed ventricular fibrillation (VF) cardiac arrest. Repeat coronary angiography demonstrated a patent LAD stent with no new angiographic abnormality. Return of spontaneous circulation was achieved after approximately one hour of resuscitation, multiple shocks and intubation.	Admitted to ICU requiring adrenaline and amiodarone infusions, mechanical ventilation and post-arrest critical care. Subsequently extubated.
Further deterioration	Developed fluid overload and ventilator-associated pneumonia. After step-down to CCU, developed further melena, severe anaemia (Hb 56 g/L) and hypotensive episodes. Esophagogastroduodenoscopy (OGD) demonstrated moderate duodenitis with mild diffuse oozing but no large ulcer or high-risk stigmata; the endoscopist considered these findings insufficient to explain the severity of bleeding.	Ventilator-associated pneumonia treated with antibiotics. Following recurrent GI bleeding, high-dose intravenous omeprazole was started with a view to conducting a CT angiogram if there were further episodes of melena.
Outcome	Suffered a further VF cardiac arrest.	Advanced life support was unsuccessful, with no return of spontaneous circulation, and the patient died.

## Discussion

This case illustrates a rare and severe presentation of adult-onset IgA vasculitis with multi-system involvement affecting the renal, GI with possible cardiac involvement. Such extensive disease is uncommon in adults and carries significant morbidity. In this discussion, we explore four key aspects of this case: potential triggers, catastrophic GI manifestations, potential cardiac complications, and rituximab as a therapeutic modality.

Uncertain etiology

The etiology of IgA vasculitis is multifactorial, involving both genetic predisposition and environmental triggers. Drugs and infections are among the most well-established precipitants [1,4]. Beta-lactam antibiotics are the most frequently implicated medications [4]. Infectious triggers have also been widely reported, with Group A *Streptococcus *identified in throat cultures in approximately 10%-30% of cases [2]. Other pathogens, including coxsackievirus, hepatitis B, hepatitis C, and parvovirus B19, have been associated with disease onset [1]. Genetic susceptibility, particularly the presence of HLA-B35 and HLA-DRB1 alleles, has also been linked to increased risk [1,2]. More recently, both COVID-19 infection and vaccination have been reported as potential immunological triggers of IgA vasculitis [6,7].

Our patient developed symptoms a few weeks after receiving the COVID-19 vaccine. A French multicenter case series of 12 patients described cases of IgA vasculitis following COVID-19 vaccination with symptom onset ranging from four to 25 days post-vaccine [6]. Additionally, a further two cases were documented in which vaccination preceded IgA vasculitis affecting the GI tract, one in a patient with prior IgA vasculitis who developed colicky abdominal pain, terminal ileitis, and later melena with hemoglobin 71 g/L; and another in a previously healthy patient with no prior history of IgA vasculitis who was admitted twice with recurrent melena, undergoing ileal resection with a biopsy confirming IgA vasculitis [7]. These cases illustrate both the potential for vaccination to precipitate recurrence in previously diagnosed patients, and to trigger severe de novo disease with GI bleeding.

While these observations highlight a possible temporal association between COVID-19 vaccination and IgA vasculitis in predisposed individuals, causality cannot be inferred, and the overall benefits of vaccination remain overwhelmingly positive.

The patient had also discontinued sulfasalazine approximately one year before this presentation, which represents an additional potential contributor in the context of his underlying autoimmune disease. Given the presence of multiple potential triggers and the observational nature of this case, it is not possible to establish whether vaccination, treatment discontinuation, or another factor precipitated the subsequent IgA vasculitis flare.

GI involvement

GI manifestations of adult-onset IgA vasculitis are well recognized, but most patients do not present with life-threatening complications. A prospective study involving histologically proven adult cases from 2013 to 2019 identified predictors of GI involvement, including generalized purpura, antecedent infection, and pre-treatment neutrophil-to-lymphocyte ratio > 3.5 [5].

In a large French multicenter retrospective study of 260 adults, GI involvement was present in 53% of patients, most frequently presenting with abdominal pain (99%) [8]. However, intestinal bleeding was only reported in 31%, while mortality was not commonly due to GI complications; GI-related deaths were primarily due to perforation or mesenteric ischemia [8]. This suggests that, at a population level, GI disease in IgA vasculitis is frequent but not universally catastrophic.

By contrast, our patient developed recurrent bloody stools, ileus, and radiological evidence of small bowel inflammation, consistent with severe enteritis. Multiple case reports describe massive intestinal bleeding as a potential complication in adult-onset IgA vasculitis, often necessitating escalation of immunosuppression [7,9-11].

One case report noted a patient with GI bleeding complicated by shock, acute kidney injury, and perforation, necessitating rituximab infusion [9]. Another case report highlighted a patient with catastrophic intestinal bleeding requiring endoscopic therapy and over 10 transfusions, with control only achieved after intravenous immunoglobulin [10]. A subsequent case highlighted diagnostic delay due to mimickers, as colonoscopy in their case initially showed terminal ileitis with elevated fecal calprotectin, leading to suspicion of Crohn’s disease, while colchicine toxicity had also been suspected [11]. They also emphasized that histological confirmation is challenging, as upper GI biopsies demonstrate vasculitis in only 5.4% of cases [11]. A case series of three patients further illustrates two cases with recurrent admission due to extensive GI involvement, with one case requiring ileal resection [7].

Taken together with our own case, these reports highlight how GI involvement in adult-onset IgA vasculitis can be severe, recurrent, and at times fatal. They also emphasize the absence of a standardized treatment escalation pathway for severe GI involvement. Finally, they underscore that oral corticosteroids are often inadequate; small bowel inflammation and impaired absorption may account for treatment failure, consistent with our patient’s initial poor response to oral therapy and subsequent improvement with intravenous steroids and rituximab.

Although this patient did not develop bowel perforation or mesenteric ischaemia, his course represented severe GI involvement, with GI bleeding, CT-confirmed small-bowel inflammation and progression to ileus requiring bowel rest, nasogastric decompression and intravenous corticosteroid therapy. The subsequent severe GI bleeding further illustrates the potential morbidity of GI involvement even in the absence of the catastrophic structural complications most frequently associated with GI mortality.

Cardiac involvement

Cardiac manifestations are exceptionally rare in IgA vasculitis. The first reported case of adult-onset disease complicated by myocardial infarction involved a 27-year-old man with relatively normal coronary angiography, establishing one of the earliest potential links between IgA vasculitis and coronary involvement [12]. Since then, a spectrum of cardiac involvement has been reported.

A published pediatric case report documented potential diagnostic mimickers where coronary artery aneurysms had initially suggested Kawasaki disease but were ultimately attributed to IgA vasculitis. Their literature search found 24 patients with cardiac involvement, of which 14 were adults. Broader cardiac involvement is described within this group, including myocarditis, dilated cardiomyopathy, pericardial effusion, and tamponade [13].

In adults, hemopericardium [14], myocardial infarction [15], and arrhythmias [16] have been described, though such reports remain sparse. One published case reported adult IgA vasculitis complicated by hemopericardium and tamponade, underscoring that cardiac involvement is not confined to ischemic-type presentations [14]. Another report described coronary vasculitis presenting with extensive thrombus formation on coronary angiography, suggesting a macrovascular mechanism as opposed to a microvascular one as suggested in our patient [15]. A comparable report detailed a patient with severe left ventricular dysfunction and arrhythmias, emphasizing that cardiac involvement may encompass both structural and electrical abnormalities [16]. In their literature search, they suggested cardiac complications were rare, having identified only 13 patients with cardiac complications, of whom only six underwent cardiac biopsy [16].

These reports highlight the significant variability in presentation and the diagnostic uncertainty; diagnosis is often inferred from treatment response or non-histological criteria.

Currently, there are no clear guidelines for managing IgA vasculitis with cardiac involvement. Some authors advocate for early escalation to immunosuppressive therapy, alongside conventional management [16]. Corticosteroids, cyclophosphamide and rituximab have all been trialed with variable outcomes [16,17].

Microvascular dysfunction has been proposed as a potential pathogenic mechanism in IgA vasculitis-related cardiac injury. In one reported case, a 55-year-old patient undergoing semi-urgent cholecystectomy was found to have acute myocarditis on cardiac MRI. Subsequent myocardial biopsy confirmed inflammatory infiltration and interstitial fibrosis consistent with IgA vasculitis, supporting a microvascular etiology of myocardial injury [18]. Potential mechanisms of cardiac injury reported in IgA vasculitis include inflammatory involvement of the coronary vasculature, myocardial inflammation and secondary thrombotic or ischaemic injury. These mechanisms provide biological plausibility for cardiac involvement during systemic IgA vasculitis [12,15,16,18].

Following the initial STEMI and LAD stenting, the patient subsequently developed ventricular fibrillation. Repeat coronary angiography demonstrated a patent LAD stent with no new obstructive coronary lesion to account for the subsequent deterioration. This raised the possibility of a non-obstructive mechanism, including inflammatory or microvascular involvement, particularly in the context of the recent severe systemic inflammatory illness. However, the patient’s pre-existing cardiovascular disease remained an important competing explanation.

Interpretation of the possible relationship between IgA vasculitis and the cardiac events requires consideration of several competing factors. The acute STEMI was associated with complete proximal LAD occlusion requiring PCI and stent placement, confirming an acute coronary event but not establishing its underlying aetiology. Conventional cardiovascular disease represents an important competing explanation given the patient’s hypertension, type 2 diabetes mellitus, pre-existing dilated cardiomyopathy and cardiac MRI findings predominantly consistent with hypertensive heart disease. Conversely, the temporal proximity to severe systemic inflammation and the rare reports of coronary and myocardial involvement in IgA vasculitis raise the possibility of an inflammatory or vasculitic contribution. In the absence of myocardial or coronary histopathology, however, direct coronary vasculitis cannot be confirmed.

Rituximab: a treatment for refractory IgA vasculitis

A systematic review of 20 studies comprising 35 patients with IgA vasculitis treated with rituximab demonstrated a favorable safety and efficacy profile. Clinical improvement was achieved in 94.3% of patients, with 74.3% maintaining remission at final follow-up. No deaths were reported, and mild adverse events occurred in only 8.6% of cases. The majority (85.7%) received rituximab due to prior corticosteroid or immunosuppressant resistance or contraindication [19]. A subsequent narrative review supported these findings, emphasizing rituximab’s role in reducing relapse frequency and corticosteroid dependence, particularly in patients with refractory renal disease [20].

In this case, escalation beyond intravenous corticosteroid therapy was prompted by ongoing severe multisystem disease, with persistent clinical and biochemical deterioration and worsening renal function despite corticosteroid treatment. Rituximab, a B-cell-depleting anti-CD20 monoclonal antibody, was selected as an additional immunosuppressive strategy in this refractory setting. However, evidence guiding the selection, sequencing, and timing of steroid-sparing or biologic therapies in severe adult IgA vasculitis remains limited, highlighting the need for clearer treatment guidance for patients with progressive organ involvement.

Rituximab has also been reported to improve IgA vasculitis-related cardiac dysfunction. In one case report, sinus bradycardia, atrioventricular block, and left ventricular systolic dysfunction successfully resolved following rituximab therapy [18]. However, the overall evidence base for managing IgA vasculitis with cardiac involvement remains limited, and treatment decisions continue to rely on extrapolation from renal and systemic disease experience.

In our patient, rituximab was administered for ongoing severe systemic IgA vasculitis prior to the subsequent cardiac event. Although clinical and biochemical improvement was initially observed, the subsequent deterioration and fatal outcome preclude conclusions regarding the effectiveness or failure of rituximab in this individual case.

Diagnostic limitations

An important limitation of this case is the absence of contemporaneous histopathological confirmation of the cutaneous and GI manifestations. The systemic diagnosis was based on the evolving clinical phenotype, including lower-limb purpura, polyarthralgia, GI involvement and renal deterioration, in the context of previous biopsy-proven IgA nephropathy. However, the cutaneous and GI manifestations were clinically attributed to IgA vasculitis rather than independently confirmed by tissue biopsy. This distinction is particularly important when interpreting individual organ involvement and the subsequent clinical course.

Although acute myocardial infarction and proximal LAD occlusion were objectively demonstrated, no cardiac biomarker, serological marker, angiographic feature or tissue biopsy specifically demonstrated coronary vasculitis. The subsequent patent LAD stent without a new obstructive lesion raised the possibility of a non-obstructive mechanism but did not establish its aetiology. The patient’s substantial pre-existing cardiovascular disease further complicated causal attribution; accordingly, the cardiac events are presented as possibly associated with IgA vasculitis rather than definitively caused by it.

Learning points

Adult-onset IgA vasculitis is uncommon and typically more severe than pediatric disease, with higher relapse and systemic complication rates. GI bleeding can signal poor prognosis and often requires early escalation beyond corticosteroids. Cardiac complications occurring during IgA vasculitis require careful differential assessment, particularly in patients with pre-existing cardiovascular disease. Rituximab may be considered in severe or refractory IgA vasculitis with progressive organ involvement; however, evidence remains limited, and treatment response should be interpreted cautiously on an individual basis. Potential triggers should be interpreted cautiously in IgA vasculitis; in this case, COVID-19 vaccination was temporally associated with disease onset, while treatment discontinuation and underlying autoimmune disease represented important potential confounders.

## Conclusions

We present a case of adult-onset IgA vasculitis with renal, GI, and possible cardiac involvement. This case underscores the emerging recognition of severe forms of the disease in adults, which carry substantial morbidity and mortality. This case particularly highlights the challenges posed by severe GI involvement with ileus, where impaired absorption may necessitate intravenous rather than oral corticosteroid therapy, and demonstrates the uncertainty involved in attributing subsequent cardiac complications to IgA vasculitis in patients with significant pre-existing cardiovascular disease.

This case highlights the challenges of managing severe adult IgA vasculitis despite treatment escalation and multidisciplinary team input, particularly in patients with substantial comorbidity, and underscores the limitations of the current evidence base for guiding management of severe disease. Furthermore, there is a pressing need to establish standardized treatment guidelines and to compare the efficacy of available immunosuppressive agents in adult IgA vasculitis. Future research should prioritize prospective studies of severe adult IgA vasculitis to define optimal treatment-escalation strategies and better characterize the frequency, mechanisms, and diagnostic features of rare GI and cardiac complications.
